# Subfatin concentration decreases in acute coronary syndrome

**DOI:** 10.11613/BM.2022.020704

**Published:** 2022-04-15

**Authors:** Mustafa Yilmaz, Mehmet Cagri Goktekin, Nevin Ilhan

**Affiliations:** 1 Department of Emergency Medicine, Firat University, School of Medicine, Elazig, Turkey; 2 Department of Biochemistry, Firat University, School of Medicine, Elazig, Turkey

**Keywords:** acute coronary syndrome, acute myocardial infarction, non-ST-elevation myocardial infarction (NSTEMI), ST-elevation myocardial infarction (STEMI), subfatin

## Abstract

**Introduction:**

We investigated the association of serum subfatin concentration and acute myocardial infarction (AMI) in patients with ST-elevation myocardial infarction (STEMI) and non-STEMI (NSTEMI).

**Materials and methods:**

In this study, patients who presented with chest pain (STEMI, NSTEMI, or non-cardiac chest pain) were included, *i.e.* 49 patients with non-cardiac chest pain (control) and 66 patients hospitalised with AMI. In the AMI group, 35 patients had NSTEMI and 31 had STEMI. Serum subfatin concentrations were determined *via* enzyme-linked immunosorbent assay (ELISA). Descriptive data on the patients and their comorbidities were recorded, and subfatin concentrations were analysed.

**Results:**

Subfatin concentrations were significantly different in the control, STEMI and NSTEMI groups (P = 0.002). In addition, subfatin concentrations were significantly lower in patients in the NSTEMI group than those in the control group (P < 0.001), but there was no significant difference between STEMI and the control group (P = 0.143). The receiver operating characteristic (ROC) analysis performed for differentiating the AMI and control groups found that subfatin had 64% sensitivity and 69% specificity, whereas troponin had 59% sensitivity and 95% specificity. In patients with AMI, the ROC analysis for differentiating NSTEMI from STEMI found that subfatin had 94% sensitivity and 41% specificity, while troponin had 65% sensitivity and 88% specificity.

**Conclusions:**

Subfatin concentrations were lower in patients without STEMI than in patients with STEMI. Subfatin concentration is associated with NSTEMI.

## Introduction

Acute coronary syndrome includes unstable angina, non-ST-elevation myocardial infarction (NSTEMI) and ST-elevation myocardial infarction (STEMI). Diagnosis and classification of acute myocardial infarction (AMI) is based on electrocardiogram findings and biochemical markers of myocardial necrosis as well as detailed examination of clinical symptoms in patients (*1*). Atherosclerotic plaque rupture, which results in partial or complete obstruction of an epicardial coronary artery, is the most common mechanism responsible for AMI. Coronary atherosclerosis is a complex, long-lasting and continuously evolving inflammatory disease (*2*, *3*).

Subfatin is an adipokine, also known as meteorin-like protein (Metrnl) and Cometin. Expressed most abundantly in white adipose tissue, in addition to the liver, spleen, muscle tissue, heart, thymus, omental adipose tissue, subcutaneous adipose tissue and interscapular adipose tissue, subfatin is a myokine that regulates inflammation and energy expenditure in adipose tissue (*4*-*6*). Experimental studies have shown that subfatin antagonises insulin resistance, and inhibits inflammation in adipose tissue through peroxisome proliferator-activated receptors gamma (PPARγ) and metabolism activation (*7*). Recently, it has been shown that subfatin is secreted from macrophages in response to local muscle damage, and supports muscle regeneration through inhibition of inflammation and induction of insulin-like growth factor 1 (*8*). Subfatin stimulates IL-4 expression, thereby increasing anti-inflammatory cytokines (*9*). Studies have found serum concentrations of subfatin to be reduced in patients with coronary artery disease (*9*, *10*).

Although subfatin has been reported to be associated with coronary artery disease and atherosclerosis, there have been few studies on the association between subfatin concentrations and AMI. In this case study, we sought to investigate subfatin concentrations in STEMI and NSTEMI in the emergency department.

## Materials and methods

### Subjects

In this case-control study a total number of 49 control subjects and 35 patients with NSTEMI and 31 with STEMI were included. The study was conducted in accordance with the Helsinki committee requirements protocol and the Ethical Committee of Fırat University, Faculty of Medicine (Date: 08/01/2019 Decision No: 12/22), and performed in the Emergency Department of Fırat University Faculty of Medicine Hospital. Sample size was calculated using the G*Power version 3.1.9.2 software (Universität Kiel, Germany). The sample size for each group was calculated to be 30 (power, 80%; P = 0.05; effect size, 0.6). Signed informed consent was obtained from all participants. After sample size was established, inclusion and exclusion criteria were determined. Patients presenting to the emergency room with chest pain that did not suggest unstable angina (retrograde burning, pain in the left arm), no ischaemic changes in electrocardiography (negative T-wave, ST-segment depression, ST-segment elevation, T inversion and left bundle branch block) and patients without elevation in troponin I concentrations at the time of admission and after 3 hours were included in the control group. Patients with acute chest pain and persistent (> 20 min) ST-segment elevation were included in the STEMI group. Patients with acute chest pain but no persistent ST-segment elevation and with transient ST-segment elevation, persistent or transient ST-segment depression, T-wave inversion, pseudonormalisation of T waves or flat T waves, or patients with normal electrocardiograms were included in the NSTEMI group if they had dynamic elevation of cardiac troponin greater than 99th percentile of healthy individuals. Patients diagnosed with AMI were divided into two groups (STEMI and NSTEMI). AMI diagnosis was established in accordance with the criteria specified in the 2020 European Society of Cardiology Guidelines (*1*). Descriptive data forms were created for patients including the following: age, sex, pain start time, weight and height, vital parameters at the time of admission to the emergency room, laboratory data (troponin, urea, creatinine and glucose) medical history including diabetes mellitus (DM), coronary artery disease (CAD), hyperlipidaemia and hypertension (HT). Data relating to the patients included in the study groups were recorded in these forms. The forms were filled based on data obtained from the patient, the patient’s relatives and the electronic information system. Samples were collected and studied between September and November 2019.

### Blood sampling

When the patients were admitted to the emergency department, blood samples were collected in test tubes containing K3-ethylenediaminetetraacetic acid (EDTA) anticoagulant (BD Vacutainer; volume 3 mL, Becton, Dickinson and Company, New Jersey, USA) for troponin measurements, and venous blood samples were collected into tubes with a separator gel (BD Vacutainer SST II Advance 8.5 mL, Becton, Dickinson and Company, New Jersey, USA) for subfatin and other biochemical measurements at the same time when the first blood samples were taken for troponin measurement. Samples for glucose, urea, creatinine and subfatin were centrifuged at 1000xg for 5 minutes after clotting. Separated serum was promptly used to measure glucose, urea and creatinine, and remaining serum aliquots were portioned to avoid repeated freeze thaw cycles, and were stored at − 70 °C until subfatin determinations were performed. In order to avoid possible loss of bioactivity, samples were analysed within 3 months from the collection and thawed only once. Before the patients were sent to relevant clinics according to their diagnosis, troponin results were taken from the information system.

### Methods

Troponin I concentrations were analysed in AQT90 flex (Radiometer Medical ApS, Copenhagen, Denmark) *via* time-resolved fluorescence measurement. The reference range considered for troponin I was 10-23 ng/L.

Serum glucose, urea and creatinine concentrations were measured at the Biochemistry Laboratory of Firat University Hospital, run on the Advia 2400 Chemistry system (Siemens Diagnostics, Tarrytown, USA). All the tests assigned by an autoanalyser were performed immediately on the serum samples obtained. The glucose, urea and creatinine concentrations were 4.2-6.4 mmol/L, 3.6-17.9 mmol/L, and 53-106 µmol/L, respectively, and they were within the reference ranges.

Concentration of serum subfatin was measured manually *via* a sandwich enzyme-linked immunosorbent assay (ELISA) (Human Meteorin-like protein ELISA Kit; Shanghai YL Biotech Co., Ltd., Catalog No: YLA3736HU, Shanghai, China) according to manufacturer’s instructions. The optical density was read using EPOCH 2 (BioTek Instruments, Winooski, USA) ELISA plate reader at the 450 nm wavelength, and the results are presented as ng/mL. The concentrations were calculated using the calibrated standard curve, which was constructed using the standards provided in the kit. The minimum detectable concentration (sensitivity) was < 0.023 ng/mL and the detection range for subfatin was 0.05-15.00 ng/mL. The intra-assay coefficient of variation (CV%) and inter-assay CV% values were less than 8% and 10%, as quoted by the manufacturer.

Under appropriate storage conditions, the loss rate was < 5% within the expiry date. Operation procedures and laboratory conditions, in particular room temperature, air humidity and incubator temperature, were strictly controlled to minimise performance fluctuations. To standardise the working environment, both the kit and serum samples were brought to room temperature before starting the study. The quality control sample was not available in the purchased kit; thus, we could not perform quality control. Standards were studied in duplicates and the r value of our standard curve was > 0.99. Our laboratory has been working on research ELISA kits for years (> 20 years). In addition, the entire assay was performed by the same operator (a biochemist).

### Statistical analysis

Data were analysed using SPSS 21.0 (IBM Corporation, Armonk, USA) and MedCalc (Version 10.1.6.0, Ostend, Belgium) software. Numerical data were expressed as median and interquartile range (IQR), and qualitative data in proportions. Body mass index (BMI) was calculated using the standard formula, body weight (kg) / height (m^2^). The Shapiro-Wilk test was used to examine if continuous variables are normally distributed. The Kruskal-Wallis test was used to compare the three groups (control, STEMI and NSTEMI). After the Kruskal–Wallis test, pairwise comparisons (control-STEMI, control-NSTEMI and STEMI-NSTEMI) were made with the *posthoc* Dunn’s test. In comparison of categorical data, the Pearson’s chi-square test was used if no more than 20% of the categories had theoretical frequencies below 5, and exact test was used above 20%. The chi-square test was followed by the *posthoc* Z-test for pairwise comparisons (control-STEMI, control-NSTEMI and STEMI-NSTEMI). The Spearman’s correlation test was used to determine the relationship between subfatin and troponin concentrations, from the onset of chest pain to admission to the emergency room. Receiver operating characteristic (ROC) curve analysis was performed using subfatin concentrations in the differentiation of control and patients with AMI, as well as STEMI and patients with NSTEMI. ROC curve analysis results were given as % specificity, % sensitivity, area under the ROC curve (AUC), P-value, 95% confidence interval (CI). For P < 0.05 statistical significance was indicated.

## Results

There was no difference in age, sex, BMI and diastolic blood pressure among the patient groups included in our study. As for basic biochemical values at the time of admission, there was no difference in urea and creatinine concentrations. However, significant intergroup differences were found in terms of troponin I, glucose and subfatin concentrations (P < 0.001, P < 0.001 and P = 0.002 respectively) (Table 1.).

**Table 1:** Patients’ basic data

	**Control**	**NSTEMI**	**STEMI**	**P**
N (Female/Male)	49 (22/27)	35 (16/19)	31(11/20)	0.639
Age	55 (35–81)	59 (36–85)	62 (40–88)	0.117
Body Mass Index	24.8 (23.9–28.9)	26.5 (25.6–29.3)	26.7 (23.9–29.3)	0.215
Troponin I (ng/L)	15 (12-18)	130 (24-355)	19 (11-34)	< 0.001
Glucose (mmol/L)	5.5 (5.2-5.7)	6.7 (5.9-10.9)	6.9 (6.4-8.1)	< 0.001
Urea (mmol/L)	12.9 (9.8-15.7)	10.7 (9.4-14.3)	12.9 (10.4–15.4)	0.380
Creatinine (µmol/L)	75 (73-82)	74 (65-87)	75 (68-84)	0.976
Coronary artery disease N (ratio)	5 (0.10)	16 (0.45)	9 (0.29)	0.001
Hypertension N (ratio)	7 (0.14)	17 (0.48)	14 (0.45)	0.001
Diabetes mellitus N (ratio)	8 (0.16)	10 (0.28)	3 (0.09)	0.127
Hyperlipidaemia N (ratio)	6 (0.12)	7 (0.20)	5(0.16)	0.627
Subfatin (ng/mL)	1.79 (1.55–2.17)	1.48 (1.32–1.70)	1.67 (1.43–2.08)	0.002
Age is expressed as median (min-max), body mass index and concentrations as median and interquartile range. Results where P < 0.05 were considered statistically significant. NSTEMI - non-ST-elevation myocardial infarction. STEMI - ST-elevation myocardial infarction.				

Pairwise comparisons of troponin concentrations with Dunn’s *posthoc* test showed significant differences in NSTEMI-control (P < 0.001) and NSTEMI-STEMI comparisons (P = 0.002), but no significant difference in STEMI-control comparison (P = 0.145).

Pairwise comparisons of glucose concentrations with *posthoc* Dunn’s test revealed significant differences in NSTEMI-control (P < 0.001) and STEMI-control (P < 0.001) pairs, but no significant difference in NSTEMI-STEMI (P = 0.933) pairs.

**Figure 1:**
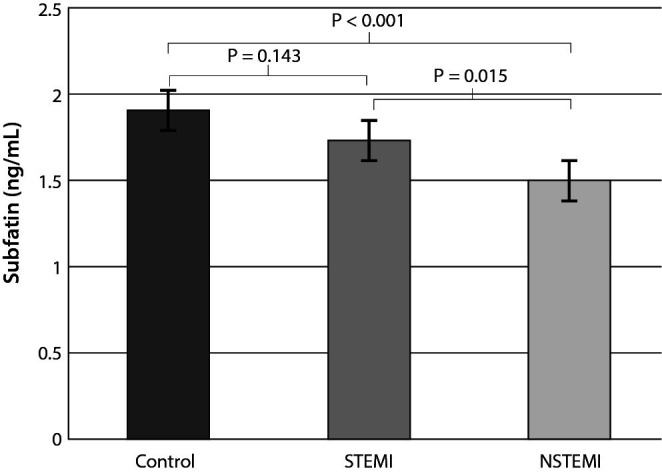
Subfatin distribution and comparison between the groups. NSTEMI - non-ST-elevation myocardial infarction. STEMI - ST-elevation myocardial infarction.

Pairwise comparisons of subfatin concentrations with *posthoc* Dunn’s test revealed statistically significant differences in NSTEMI-control (P < 0.001) and NSTEMI-STEMI (P = 0.015) comparisons, but no significant difference in STEMI-control (P = 0.143) comparison (Figure 1).

When patients with AMI were examined for concomitant chronic diseases, no significant difference was detected in subfatin concentrations between patients with and without DM (P = 0.657), HT (P = 0.662), CAD (P = 0.602) and hyperlipidaemia (P = 0.727). There was also no difference between sexes (P = 0.445) (Table 2).

**Table 2:** Concentrations of subfatin by sex and chronic comorbidity

		**Subfatin (ng/mL)**	**P**
Sex	Female (N = 27)	1.51 (1.12-2.23)	0.445
	Male (N = 39)	1.57 (0.95-2.39)	
Hypertension	Yes (N = 31)	1.54 (1.03-2.39)	0.662
	No (N = 35)	1.50 (0.95-2.34)	
Diabetes mellitus	Yes (N = 13)	1.57 (1.12-2.39)	0.657
	No (N = 53)	1.50 (0.95-2.23)	
Coronary artery disease	Yes (N = 25)	1.55 (1.11-1.29)	0.602
	No (N = 41)	1.50 (0.95-2.23)	
Hyperlipidaemia	Yes (N = 12)	1.65 (1.11-2.39)	0.727
	No (N = 54)	1.52 (0.95-2.34)	
Subfatin concentrations are expressed as median (min-max). Results where P < 0.05 were considered statistically significant.			

In patients diagnosed with AMI (NSTEMI and STEMI) (N = 66), no significant correlation was found between subfatin concentrations and time from the onset of chest pain to emergency room admission (r = − 0.20, P = 0.105), BMI (r = − 0.24, P = 0.054), blood glucose concentrations (r = 0.09, P = 0.457), troponin concentrations (r = − 0.06, P = 0.663) and urea (r = 0.07, P = 0.577) and creatinine concentrations (r = 0.02, P = 0.873).

**Figure 2:**
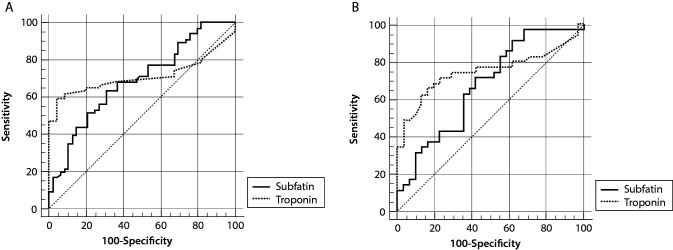
ROC analysis chart for the use of subfatin and troponin in differential diagnosis. A) ROC curve for discriminating AMI from control, B) ROC curve for discriminating NSTEMI from STEMI. AMI – acute myocardial infarction. NSTEMI - non-ST-elevation myocardial infarction. STEMI - ST-elevation myocardial infarction. ROC - receiver operating characteristic.

The ROC analysis for differentiating AMI and control group found that subfatin had 64% sensitivity and 69% specificity, whereas troponin had 59% sensitivity and 95% specificity. In patients with acute coronary syndrome, the ROC analysis for differentiating NSTEMI from STEMI found that subfatin had 94% sensitivity and 41% specificity, whereas troponin had 65% sensitivity and 88% specificity (Table 3 and Figure 2).

**Table 3:** Receiver operating characteristic (ROC) analysis data for the use of subfatin and troponin in the differential diagnosis.

		**Cut Off**	**AUC**	**95% Cl**	**Sensitivity (%)**	**Specificity (%)**	**LH(+)**	**LH(-)**	**P**	P*
**A**	Subfatin (ng/mL)	≤ 1.69	0.690	0.60-0.77	64	69	2.08	0.52	< 0.001	0.757
	Troponin (ng/L)	> 0.02	0.713	0.62-0.79	59	95	14.48	0.43	< 0.001	
**B**	Subfatin (ng/mL)	≤ 1.81	0.705	0.58-0.81	94	41	1.59	0.14	0.001	0.419
	Troponin (ng/L)	> 0.07	0.750	0.66-0.87	65	88	5.18	0.40	< 0.001	
*Comparison of subfatin and troponin ROC analysis. A) ROC curve for discriminating AMI from control. B) ROC curve for discriminating NSTEMI from STEMI. LH(+) - Positive likelihood ratio. LH(−) - negative likelihood ratio. AMI – acute myocardial infarction. NSTEMI - non-ST-elevation myocardial infarction. STEMI - ST-elevation myocardial infarction. AUC - area under the curve. Cl - confidence interval.										

## Discussion

Subfatin concentrations were lower in patients without STEMI than in patients with STEMI. No significant difference was found between STEMI and patients in the control with non-cardiac chest pain group. Studies on the relationship of subfatin with Type 2 DM (T2DM) or CAD are few; however, the concentration of this adipokine reportedly changes in obesity and metabolic syndrome associated with CAD and T2DM (*11*). Lee *et al.* reported low subfatin concentrations in newly diagnosed patients with T2DM (*12*). In addition, subfatin has been reported to cause increased expression of anti-inflammatory genes such as interleukine (IL) - 10, transforming growth factor beta (TGF-beta) and decreased expression of pro-inflammatory genes such as tumor necrosis factor alpha (TNF alpha), interferon-gamma and IL-1 beta. Atherosclerotic plaque rupture is the most common mechanism responsible for AMI. Inflammation plays an important role in the development and progression of atherosclerosis (*13*). Dadmanesh *et al*. have reported an association between subfatin and coronary artery disease and atherosclerosis, and found significantly lower concentrations of subfatin in patients with CAD and DM. They have found 68% sensitivity and 64% specificity in differentiating healthy control group from those with T2DM; and 68% sensitivity and 67% specificity in differentiating healthy control group from those with CAD (*10*). However, a meta-analysis published in recent years found no significant correlation between changes in Metrnl concentrations and Type 2 DM and CAD risk than healthy control subjects; however, there was an inverse relationship between circulating Metrnl and BMI > 25 (*6*). Results from experimental studies have shown that subfatin can reduce doxorubicin-induced cardiotoxicity by activating the cyclic AMP/protein kinase A/Sirtuin1 pathway, and reduce cardiomyocyte apoptosis caused by ischaemia/reperfusion injury through activation of AMP-activated protein kinase/p21-activated kinase 2 signalling (*14*, *15*). Cai *et al.* showed that low serum Metrnl concentrations in elderly patients with congestive heart failure were associated with weight loss and the severity of cardiac dysfunction (*16*). Our results demonstrated that the sensitivity and specificity was 64% and 69%, respectively, when differentiating patients with AMI (STEMI and NSTEMI) from those without AMI, whereas the sensitivity was 94% and specificity was 41% when in differentiating STEMI from NSTEMI patients. However, troponin concentrations had superior sensitivity and specificity in the differential diagnoses. Subfatin concentrations may be reduced in patients with NSTEMI owing to the relatively longer and irreversible cellular damage.

Lee *et al.* reported that there was no significant relationship between subfatin and glucose concentrations (*12*). Similarly, Pellitero *et al*. reported that there was no sex difference in subfatin concentrations, and there was no difference between normoglycemic patients with obesity and patients with type 2 diabetes (*17*). Similarly, our study found no difference in subfatin concentrations between those with DM and without DM. There was also no significant correlation between subfatin and blood glucose concentrations. This may be attributable to the small number of patients with DM (N = 21) included in our study, and the fact that these patients were under anti-diabetic treatment.

Previous experimental studies have shown increased subfatin concentrations in obese mice (*5*). However, conflicting results have been achieved on BMI in studies with humans. Lee *et al.* reported that there was no significant relationship between subfatin and BMI (*12*). Pellitero *et al.* found in their study that subfatin concentrations were lower in patients with obesity than in patients with normal weight. Although it increased significantly 12 months after obesity surgery, post-obesity surgery levels remained below that of patients with normal weight (*17*). However, Fens *et al.* has found an inverse relationship between circulating Metrnl and BMI (*6*). In our study, there was no difference between patients in terms of BMI. There was also no significant correlation between BMI and subfatin concentrations.

There are some limitations in our study. Among these are the lower subfatin concentrations in patients with AMI; since patients’ subfatin concentrations before AMI could not be detected, it could not be fully understood whether the decrease in subfatin concentrations during AMI was completely owing to acute myocardial necrosis. The second limitation is the fact that subfatin concentrations were not measured in patients after treatment. Another limitation is the fact that data on chronic diseases were solely derived from patients’ verbal explanation and we could not rely on subjective data. Drugs used by the patients were not classified in the present study. Drugs used by patients may also affect subfatin concentrations.

In conclusion, we found that subfatin concentrations, known to be associated with CAD and atherosclerosis, also decreased in AMI. This decrease was more pronounced in patients with NSTEMI. However, compared to the increase in troponin concentrations in patients with NSTEMI, the decrease in subfatin concentrations did not have adequate specificity and sensitivity to be considered for use in NSTEMI diagnosis.
